# Evaluation of Retinal Structure and Optic Nerve Function Changes in Multiple Sclerosis: Longitudinal Study with 1-Year Follow-Up

**DOI:** 10.1155/2021/5573839

**Published:** 2021-06-17

**Authors:** Riwanti Estiasari, Adisresti Diwyacitta, Muhammad Sidik, Ni Nengah Rida Ariarini, Freddy Sitorus, Saraf Shafa Marwadhani, Kartika Maharani, Darma Imran, Reza Aditya Arpandy, David Pangeran, Manfaluthy Hakim

**Affiliations:** ^1^Department of Neurology, Cipto Mangunkusumo General Hospital, Universitas Indonesia, Jakarta, Indonesia; ^2^Department of Ophtalmology, Cipto Mangunkusumo General Hospital, Universitas Indonesia, Jakarta, Indonesia

## Abstract

**Background:**

Multiple sclerosis (MS) is an autoimmune disease characterized by inflammation and demyelination of the central nervous system which often involves the optic nerve even though only 20% of the patients experience optic neuritis (ON).

**Objective:**

This study aims to compare the retinal structure and optic nerve function between patients with MS and healthy controls (HCs), evaluate optic nerve alterations in MS over 1-year follow-up, and analyze its correlations with disease duration, number of relapses, degree of disability, and different subtypes.

**Methods:**

This is a prospective cohort study involving 58 eyes of MS patients. Optic nerve function was evaluated with best-corrected visual acuity (BCVA), contrast sensitivity, and P100 latency, while the retinal structure was evaluated from the GCIPL and RNFL thickness measured with optical coherence tomography (OCT) and fundus photography.

**Results:**

The MS group had lower BCVA (*p*=0.001), contrast sensitivity (*p* < 0.001), mean GCIPL thickness (*p* < 0.001), and mean RNFL thickness (*p* < 0.001) than HC. At 6 and 12 months of observations, GCIPL and RNFL (nasal quadrant) of MS patients decreased significantly (*p*=0.007 and *p*=0.004, respectively). Disease duration and the number of relapses correlated with delayed P100 latency (*r* = −0.61, *p* < 0.001 and *r* = −0.46, *p*=0.02). GCIPL and RNFL in the SPMS subtype were thinner than in RRMS.

**Conclusions:**

The retinal structure and optic nerve function of MS patients are worse than those of normal individuals. GCIPL and RNFL thinning occurs at 6 and 12 months but do not correlate with disease duration, the number of relapses, and degree of disability.

## 1. Introduction

Multiple Sclerosis (MS) is a chronic immune-mediated disease of the central nervous system affecting 2.5 million people globally [1]. The prevalence of MS in Indonesia is 0–5 per 100.000 individuals with the highest incidence in women aged 20 to 40 [2–4]. Although considered a rare disease, MS is the leading cause of nontraumatic neurological disability in the young population [1]. MS data in Indonesia are rarely reported. A previous study in Indonesia demonstrated that the clinical characteristics of MS which were generally similar to those of MS patients in Western countries. The most subtypes found in Indonesia are also RRMS [2].

Inflammatory and demyelinating pathology in MS frequently involves the visual pathway [5]. Despite only 20% of MS patients showing optic neuritis (ON), an autopsy study revealed that 94–99% of MS patients have detectable optic nerve lesions even in the absence of any visual symptoms [6, 7]. Given its susceptibility to damage in the early stage of the disease, visual measures have been a focus of recent studies to evaluate the progression of MS [8–10].

Numerous tools and techniques have been developed to portray the structure and function of the optic nerve that can provide clinically meaningful information of MS [10–12]. The structural composition of the retina can be observed using Optical Coherence Tomography (OCT), while the function may be evaluated through the Visual Evoked Potential (VEP) test [8, 11]. According to The 25-Item National Eye Institute Visual Functioning Questionnaire (NEI-VFQ-25) and 10-Item Neuro-Ophthalmic Supplement, the most common visual symptoms in MS include a decrease in visual acuity and contrast sensitivity [12]. The latter can be observed using the Pelli-Robson charts.

The pathogenesis of optic neuritis is not well understood. A recent study suggests that axonal damage, not related to demyelination, is the major contributor to permanent visual loss [13]. It is reflected in the thickness of the retinal nerve fiber layer (RNFL), the innermost retinal layer which comprises nonmyelinated axons of the retinal ganglion cells (RGCs) [13]. OCT offers 75% sensitivity and 81% specificity to monitor axonal and neuronal loss in MS in terms of RNFL thickness [14]. Studies showed that RNFL thickness is lower in MS patients than that in normal individuals and would decrease by 10–40 mm within 3–6 months after an acute ON episode [11, 15, 16]. Recent findings suggest that the ganglion cell inner plexiform layer (GCIPL) is more accurate in depicting the progression of MS. It also demonstrates a better correlation with visual dysfunction as it is less sensitive to neuroinflammation [17, 18].

VEP serves as an approach to assess the function of the optic nerve by recording electrical changes following stimulation [11]. The sensitivity and specificity of VEP in MS are 60.7% and 80.5%, respectively. Another study reported higher VEP sensitivity of 90% [19]. A typical finding of MS during VEP measurement is prolonged P100 latency regardless of any prior episode of ON [11].

Previous studies have investigated the association between the retinal structure and optic nerve function with disease duration, number of relapses, subtype, and degree of disability in MS. As discussed by Balk et al., the thinning of RNFL and GCIPL is influenced by disease duration and occurs predominantly in the early stage of the disease [20]. In another study, a significant association was only found between the temporal RNFL and disease duration [21]. The progression of MS seems to follow relapse, but no evidence proves that relapse causes progression [22]. A multicenter study showed that RNFL thickness is lower in secondary progressive type (SPMS) than relapsing-remitting type (RRMS) [23]. RNFL, GCIPL, and contrast sensitivity are also known to affect the Expanded Disability Status Scale (EDSS) score with GCIPL having the strongest association with EDSS [24–26]. RNFL thickness lower than 88 *μ*m is associated with a threefold increased risk of EDSS progression in 3 years [27].

In our previous study, we found that GCIPL, RNFL, and visual acuity of patients with Neuromyelitis Optica Spectrum Disorders (NMOSD) are lower than those of patients with MS [28]. The three parameters were also inversely correlated with disease duration, while the number of relapses was the only parameter that showed no association with other variables. The limitation of this study is the lack of healthy controls.

Given the information, the aims of this study are (1) to compare the retinal structure and optic nerve function between multiple sclerosis patients and healthy individuals, (2) to evaluate the alteration of retinal structure and optic nerve function of MS patients in the 1-year follow-up, and (3) to explore the association between visual parameters with disease duration, the number of relapses, and degree of disability in MS.

## 2. Materials and Methods

### 2.1. Design and Participants

This was a prospective cohort study with a duration of 1 year, starting from September 2018 to April 2019. Participants were recruited from the Neurology clinic at Dr. Cipto Mangunkusumo General Hospital using a consecutive nonrandom sampling method. Eligible patients must sign a written consent before participating in the study. This research was approved by the Ethics Committee of the Faculty of Medicine Universitas Indonesia and was funded by Hibah PITTA 2018 DRPM Universitas Indonesia (PITTA/214/FK/2018).

A total of 102 eyes from 32 patients with MS and 22 normal individuals were evaluated at baseline, 6 months, and 12 months. We used our previous research data of 110 normal individuals (220 eyes) for VEP controls [28]. All eyes were classified into three groups: multiple sclerosis (58 eyes), healthy control for OCT (44 eyes), and healthy controls for VEP (110 eyes).

### 2.2. Inclusion and Exclusion Criteria

Inclusion criteria were (1) age 18–60 years old, (2) providing a written consent containing all tests included in the study, and (3) meeting the diagnostic criteria for MS (exposed group) or having best-corrected visual acuity (BCVA) of 20/20 (healthy control).

Exclusion criteria were (1) experiencing ON within 6 months before enrollment, (2) having other types of optic neuropathy (ischemic, infection, hereditary, compression/infiltration, toxic, and trauma), (3) having other ophthalmological conditions (refractive errors >6 diopters (D), glaucoma), (4) having diabetes mellitus, grade II hypertension, or obesity (BMI ≥40 kg/m^2^), and (5) having a history of taking drugs with ocular side effects, such as hydroxychloroquine and ethambutol.

### 2.3. Data Collection

Anamnesis, physical examination, and neurological examination were performed on all participants. Confounding illnesses and conditions were excluded during history taking and physical examination. Intraocular pressure (IOP) was measured on both eyes with a Schiotz tonometer, using pantocaine 0.5% as an anesthetic. Demographic data (sex and age), disability status (EDSS), and the number of relapses were recorded. We analyze the optic nerve structure using OCT and retinal fundus image, and for function, we used the VEP (P-100 latency).

Best-corrected visual acuity (BCVA) was assessed using the Snellen chart, and contrast sensitivity was measured monocularly using the Pelli-Robson chart. All measurements were repeated at 6 and 12 months. Some of the data were obtained from the patients' medical records since BCVA, contrast sensitivity, OCT, VEP, and fundus photography were part of our routine clinical evaluations at the clinic. For the VEP measurement of healthy control, we used secondary data from our previous research by Wijaya et al. [29].

#### 2.3.1. Optical Coherence Tomography and Retinal Fundus Images

We used Cirrus HD OCT 5000 with optic disc cube 200 × 200 protocol and macular cube 512 × 128 protocol. RNFL thickness measurement was done by a certified optician. Participants were scanned in pupillary dilation which was obtained with tropicamide 1%. The fundus photograph was taken for each eye using the Kowa VX-10 fundus camera. The resulting images were masked and reviewed by 2 experts based on the agreed operational definition. If any discrepancy happened between the 2 reviewers, a third person would make the decision.

#### 2.3.2. Visual Evoked Potential

VEP was measured monocularly on both eyes with Caldwell Sierra Summit and 24 inch LCD ViewSonic VT2216-L monitor which had been calibrated digitally. The VEP was interpreted by a neurophysiologist.

### 2.4. Statistical Analysis

In the descriptive analysis, quantitative variables are presented in mean and standard deviation for normally distributed data or median and range for nonnormally distributed data. Statistical analysis was carried out using SPSS version 20.0. Numerical variables are tested for normality before proceeding with further analysis. In variables of which the participants were measured multiple times, data were analyzed using repeated ANOVA if distributed normally. Otherwise, the Friedman test was used. We used the T-independent test to compare mean scores of the same variable from two different groups for normally distributed data or the Mann–Whitney for nonnormally distributed data. To test the correlation, we used Pearson's correlation coefficient for normally distributed data, otherwise Spearman's correlation formula. To assess qualitative variables and determine the agreement between two reviewers, we used the kappa coefficient. Kappa coefficient is acceptable if the value is >0.8 [30]. Statistical significance was established at *p* < 0.05.

## 3. Results

### 3.1. Baseline Characteristics

Initially, 32 MS patients were recruited in the study. Four eyes were excluded due to severe nystagmus and the other four had a high refractive error of ≥6 diopters, yielding a total of 58 eyes. Besides, there is one missing data on GCIPL in healthy controls for OCT (HC OCT) due to recording error. For VEP healthy control (HC VEP), we used data from our previous research by Wijaya which included 110 normal participants (220 eyes) [29]. The baseline characteristics of both MS and healthy controls are depicted in Table 1. In general, all groups are homogeneous, except for the sex ratio between the MS group and HC VEP (*p* < 0.001). The ratio of male: female is 1 : 5 with ethnic distribution, Javanese (26.7%), Chinese descent (23.3%), Sundanese 10%, Manado, Minang, Betawi 6.7%, and Batak 3%. There was no difference in the percentage of optic neuritis occurrences between these ethnic groups.

We identified 58 eyes at the entry visit (baseline), but only 34 eyes could be evaluated at 6 months. One participant was excluded for being 60 years by the time of the second measurement, and the rest were lost to follow-up. At 12 months, only 10 patients (20 eyes) returned for the last measurement. One patient (2 eyes) died, and 6 patients (12 eyes) could not make it to the hospital.

### 3.2. Comparison of the Retinal Structure and Optic Nerve Function between MS Patients and Healthy Controls

We found that the baseline retinal structure and optic nerve function in MS are lower than those in healthy controls, marked by significantly thinner GCIPL and RNFL (in all sectors except the nasal quadrant) and longer P100 latency. Optic atrophy was only seen in the MS group with a percentage of 42.6%. See Table 2.

### 3.3. Alteration of Retinal Structure and Optic Nerve Function in MS Patients at 6 and 12 Months

Table 3 summarizes the functional evaluations obtained at baseline, 6 months, and 12 months. No significant changes were observed in BCVA and contrast sensitivity from all time. Meanwhile, P100 latency was significantly longer at 12 months compared to 6 months (124.43 ± 19.13 vs 110.07 ± 19.97, *p* < 0.001) and baseline (124.43 ± 19.13 vs 116.63 ± 19.19, *p*=0.002).

At baseline, 23 out of 58 eyes presented with optic atrophy on their fundus photographs. Six months later, among 34 eyes being scanned, six previously normal eyes worsened into optic atrophy, two of which had a history of ON, and another two never had ON nor relapses. These findings were captured from four female patients aged 27–47 years who had been diagnosed with RRMS for 6 months to 6 years. Their EDSS score ranged from 1.0 to 6.0. On the other side, one eye exhibited recovery from optic atrophy which was obtained from a 22-year-old female in the absence of prior ON. She had been diagnosed with RRMS for 7.5 years and experienced 3 relapses. Her EDSS score was 2.5. At 12 months, one previously normal eye developed optic atrophy which was observed from a 29-year-old female with RRMS and no history of ON. She had been diagnosed with MS for 6 years with 6 relapses and an EDSS score of 1.5.

**Figure 1:**
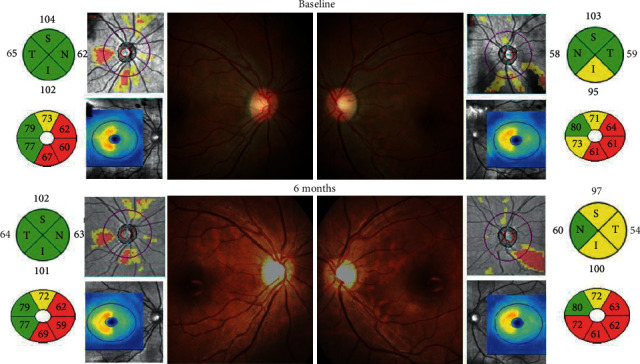
28-year-old female with SPMS. The RNFL and GCIPL become thinner, optic nerve head appeared paler, but contrast sensitivity was stable during the 6-month observation.

As seen in Table 4, the present study shows a decrease in GCIPL thickness in MS patients over 1 year with the most prominent change observed between the baseline and the 12-month evaluation (70.89 ± 10.60 vs 67.95 ± 11.12, *p*=0.01). Statistically significant thinning was found in all quadrants, except the superior and superotemporal quadrant (Figure 1). In the superonasal quadrant, a significant difference was found between the 6- and 12-month evaluations (69.88 ± 12.65 vs 68.05 ± 12.90, *p*=0.04). In the inferonasal quadrant, the differences were noted between the 0- and 6-month evaluations (70.28 ± 12.74 vs 68.65 ± 12.65, *p*=0.04) and 0- and 12-month evaluation (70.28 ± 12.74 vs 67.41 ± 13.75, *p*=0.01). In the inferior and inferotemporal quadrant, a significant difference was observed between the 0- and 12-month evaluations (69.35 ± 11.06 vs 66.45 ± 11.68, *p*=0.046; 71.83 ± 10.12 vs 68.36 ± 10.49, *p*=0.01).

In contrast to GCIPL, a significant difference in RNFL thickness was only seen in the nasal quadrant between the 0- and 12-month evaluations, where the thickness increased at 12 months after a slight decrease at 6 months.

### 3.4. Correlation Analysis of Visual Parameters, Disease Duration, and Number of Relapses

The number of relapses showed a negative correlation with GCIPL thickness of the superonasal quadrant (*p*=0.03, *r* = −0.29), inferonasal (*p*=0.03, *r* = −0.30), and mean RNFL thickness (*p*=0.04, *r* = −0.28). EDSS score showed a negative correlation with mean GCIPL, the superonasal, inferonasal, and inferior quadrant thickness. In addition, EDSS was inversely correlated with the RNFL thickness of the inferior quadrant (*p*=0.01, *r* = −0.34).

We found that the rates of change on P100 latency, RNFL thickness, and GCIPL thickness throughout the study were relatively stable. In correlation analysis, the rate of change on P100 latency was shown to have a negative correlation with disease duration (*r* = −0.61, *p* < 0.001) and the number of relapses (*r* = −0.46, *p*=0.02).

### 3.5. Comparison of the Retinal Structure and Optic Nerve Function in MS Subtypes

Of all 32 MS participants included in this study, 25 patients (78%) belonged to the relapsing-remitting subtype (RRMS) and 7 patients (22%) to the secondary progressive subtype (SPMS). No significant difference was observed between RRMS and SPMS in terms of visual acuity, contrast sensitivity, P100 latency, and fundus image. GCIPL thickness on SPMS eyes was significantly thinner compared to RRMS eyes on average and in distinctive quadrants (see Table 5). Likewise, RNFL thickness was significantly lower in SPMS than RRMS, except for the nasal quadrant.

### 3.6. Comparison of Visual Parameters Based on the History of Optic Neuritis

In the present study, ON was reported in 17 eyes (MS-NO), while the other 41 eyes had no history of ON (MS-NNO). In MS-NO, no significant alteration was detected in almost all visual parameters over 1-year follow-up (visual acuity, contrast sensitivity, RNFL, P100 latency, and fundus image). The only meaningful change was observed in the inferonasal GCIPL thickness between 0 and 6 months, where it increased significantly (67.62 ± 16.99 vs 70.33 ± 17.23, *p*=0.02). In terms of fundus photographs, the results of the MS-NO group were relatively stable throughout the study. There were initially 8 eyes with normal fundus images and 7 eyes with optic atrophy at baseline. At 6 months, 2 previously normal eyes developed optic atrophy.

In contrast to MS-NO, we observed significant differences of GCIPL as well as of P100 latency in MS-NNO throughout 1 year. P100 latency lengthened significantly at 12 months compared to baseline (128.20 ± 18.96 vs 113.13 ± 20.25, *p*=0.001) and 6 months (128.20 ± 18.96 vs 114.70 ± 17.44, *p*=0.02). With respect to GCIPL, significant changes were observed (1) between 0 and 6 months in the inferotemporal quadrant (73.41 ± 8.67 vs 70.52 ± 8.97, *p*=0.04) and superotemporal quadrant (72.17 ± 8.36 vs 69.56 ± 8.72, *p*=0.04) and (2) between 0 and 12 months in the inferonasal quadrant (71.12 ± 11.20 vs 65.19 ± 10.65, *p*=0.024), inferior quadrant (70.80 ± 9.70 vs 65.31 ± 9,10, *p*=0.041), and mean GCIPL (72.22 ± 9.26 vs 66.81 ± 8.9, *p*=0.011).

In terms of fundus photographs, the ratio of normal and optic atrophy in the MS-NNO tended to decrease over time. At the start, 23 eyes had a normal fundus appearance while 16 eyes had optic atrophy. At 6 months, 2 patients (4 eyes) developed optic atrophy (see Table 6). They were 47- and 27-year-old patients with RRMS. Their disease duration, number of relapses, and EDSS score were as follows: 6 months, 3 years; no relapse, 6 relapses; 1.0, 6.0. In contrast, there was 1 eye that recovered from optic atrophy to normal fundus. This eye was scanned from a 22-year-old female RRMS patient with 7.5 years of disease duration and 2.5 of EDSS score. At 12 months of evaluation, 1 eye developed optic atrophy which was evaluated from a 29-year-old female with RRMS with 6 years of disease duration, 6 previous relapses, and 1.5 of EDSS score.

## 4. Discussion

### 4.1. Visual Parameters in MS and Healthy Controls

Our study shows that MS patients have significantly lower BCVA, contrast sensitivity, GCIPL, RNFL, and longer P100 latency than healthy controls which are consistent with previous studies [12, 24, 31]. GCIPL thinning among MS patients may be caused by a microscopic optic nerve inflammatory process, where the retinal ganglion cells (RGCs) are at greater risk for neurodegeneration. Thus, rates of GCIPL thinning are faster in patients exhibiting evidence of disease activity [32].

In our study, RNFL thinning was apparent in every sector except the nasal quadrant, corroborating a study by Pillay et al. [31] which suggests that the nasal quadrant is most resistant to RNFL loss. On the other hand, the temporal quadrant is most susceptible to damage and may be the earliest and dominant manifestation of visual dysfunction [33]. While the exact cause of this phenomenon is unclear, one plausible explanation is that the temporal RNFL quadrant is made up primarily of small-sized parvocellular axons inside the papillomacular bundle. Whether the smaller axons are more prone to damage is unknown, but they may have a less efficient remyelination process than larger axon [34]. Moreover, according to previous studies, temporal RNFL has a different developmental pattern from other quadrants. The expansion of the sclera during this development causes the nerve fibers to be distributed over a wider surface area, resulting in the early thinning of temporal RNFL. This pattern does not occur in the nasal quadrant where the thickness is higher [35].

We also found significantly delayed P100 latency in MS patients, as has been reported in previous studies [11, 36, 37]. Compared to MRI, VEP is more sensitive in detecting optic nerve demyelination but not sensitive enough to confirm MS diagnosis. In suspected cases of MS without evidence of ON, delayed latency of P100 in VEP may indicate optic nerve demyelination and suggest the incident of MS [38].

Compared with healthy individuals, MS patients have a significantly higher rate of optic atrophy. In certain cases, optic atrophy may result from a previous severe inflammation that occurs diffusely or temporally [39].

### 4.2. Evaluation of Visual Parameters in MS Group over 1 Year

As discussed by Chatziralli et al., visual acuity is restored almost completely within 6 months after an ON episode [11]. Similarly, contrast sensitivity showed equal improvement in 6–9 months after the conduction block caused by edema and inflammation has cleared up [40]. In the present study, we excluded patients with a history of ON within 6 months prior to enrollment which explains why we did not obtain a significant difference in visual acuity and contrast sensitivity in the MS group throughout the study.

The VEP results of our MS participants are in line with previous studies that reported lengthening of P100 latency 3 months after an acute episode and lasted for 1 year [40, 41]. Another study suggests that VEP abnormality may still be detected after ≥1 year but the rates of deceleration will decrease [42].

In our analysis, the average value of GCIPL along with the inferior, inferotemporal, inferonasal, and superonasal quadrant in MS patients decreased significantly over 1 year. This finding supported other studies by Pillay [31] and Rebodella [43], who also reported that the supero- and inferonasal quadrants had the highest abnormality rate. As discussed by Shi et al. [44], the thinning zone occurs predominantly at nasal 1.98 mm and inferior 0.42 mm from the fovea with a circular area (diameter = 1 mm), called the M zone (for MS). The M zone may demonstrate the involvement of papillomacular bundle in the parvocellular pathway. It is true, according to pathological studies, that smaller axons are damaged in MS. Moreover, mitochondrial dysfunction also played a role in MS and the location of the M zone is similar to that of mitochondrial optic neuropathy [44].

In this study, we only found a significant RNFL thinning in the nasal quadrant between 0- and 12-month measurement which is contradictory to previous studies [31, 45]. In a study by Garcia-Martin et al. [45], maximal RNFL loss was found in the superonasal and inferotemporal quadrant. In contrast, an improvement was observed in the superior and nasal quadrant between 0 and 6 months and 6 and 12 months, respectively. This increase is possibly due to astrogliosis which results in a false thickening of RNFL [18]. Thus, astrogliosis can impede the accuracy of OCT in measuring RNFL thickness.

We also observed an increase in the proportion of optic atrophy at 6 and 12 months. Regardless of the high dropout rate, at the 6 months alone, there were 6 eyes that developed optic atrophy. Hence, it is safe to say that the increased proportion was due to the progression of the disease itself, not attrition bias. The optic disc is the entry point of the optic nerve which is composed of numerous axons. In MS, axonal degeneration is responsible for the development of optic atrophy observed during funduscopy [39].

### 4.3. Correlation between Visual Parameters, Disease Duration, Number of Relapses, and Degree of Disability in MS Patients

Our analysis showed that GCIPL (superonasal, inferonasal quadrant) and mean RNFL had a negative correlation with the number of relapses. Likewise, GCIPL (mean, superonasal, inferonasal, inferior quadrant) and RNFL (inferior quadrant) demonstrated a negative correlation with the EDSS score as reported by previous studies [26, 32]. Pillay et al. [31] reported that RNFL < 88 *μ*m is associated with a threefold increase in the EDSS score in 3 years. Unlike previous studies, we found no correlation between OCT measures and disease duration [11, 21]. However, P100 latency was significantly correlated with disease duration and the number of relapses. Bennetto et al. [22] also reported a similar finding but his study needs further statistical analysis to make a conclusion. According to the London Ontario cohort study, higher relapse frequency in the first 2 years is associated with a lower EDSS score [46].

Over 1-year evaluation, we found that GCIPL and RNFL alteration occurred at a more constant rate than P100 latency, both in the first 6 months or in the following months. Our findings appear contradictory with a study by Henderson et al. [21] which concluded that RNFL thinning does not follow a linear progression. They suggest that RNFL loss occurs faster in the early course of the disease when subclinical inflammatory demyelination takes place.

### 4.4. Differences of Visual Measures between MS Subtypes

We observed a significant difference in GCIPL and RNFL thickness between RRMS and SPMS at baseline. This finding indicates that neurodegeneration, marked by axonal and ganglion cell loss, occurs more rapidly in MS with progressive phase, regardless of disease duration, age, and previous ON history [47]. However, a different finding was reported by Balk et al. [20], in which a significant difference of RNFL (but not GCIPL) was observed between RRMS and progressive MS over 2 years. In other words, even though neuronal and axonal injury occurs during a relapse episode, only a few continue to deteriorate in the progressive phase [21]. In RRMS, the remitting phase provides the opportunity for the remyelination process to take place. However, in SPMS, demyelination or axonal degeneration happens without the remitting phase, supporting the fact that the damage caused in SPMS is more severe. Therefore, it can be seen at the beginning that the structure and function of the optic nerve in SPMS are clinically poorer than RRMS.

### 4.5. History of Previous ON Influencing the Visual Parameters

We also performed an adjusted analysis based on the patient's history of ON. In the MS-NO subgroup, GCIPL appeared to thicken over 12 months but a significant difference was found only in the inferonasal quadrant. Other parameters only showed subtle changes which may be due to the participants being in the remission phase and having no acute episode during the study [31]. Furthermore, if we look at the whole sample regardless of previous ON, significant thinning was actually seen in every quadrant. Therefore, it can be concluded that the relatively stable visual measures among the MS-NO subgroup were because of insufficient sample size, assuming that participants who stayed in the study had thicker GCIPL than those who dropped out.

In contrast to MS-NO, the significant differences of OCT measures in the MS-NNO subgroup were possibly due to axonal loss resulting from previous subclinical ON that left a profound manifestation on the GCIPL [31]. Delayed P100 latency in this group corresponds with Pillay et al. who reported similar findings in eyes without a history of ON at 6 months [11]. In suspected MS cases, abnormality in VEP measurement illustrates the demyelinating process of the optic nerve and provides information about MS development [38].

## 5. Conclusion

In summary, this study showed that MS patients have lower visual acuity and contrast sensitivity, thinner GCIPL and RNFL thickness (except in the nasal quadrant), and longer P100 latency compared with healthy individuals. GCIPL and RNFL thinning occurs at 6 and 12 months but does not correlate with disease duration, the number of relapses, and EDSS score. The thinning of GCIPL from 0 to 12 months is more prominent compared with RNFL, and it is shown in the inferonasal, inferior, and inferior temporal quadrant. The thinning of GCIPL and RNFL is worse in SPMS than in RRMS.

### 5.1. Strength and Limitation

In Indonesia, this is the first longitudinal study of multiple sclerosis in which the data on MS are still scarce. We recruited healthy controls with matching age and sex with the MS group. The optic nerve was evaluated in various parameters to give more comprehensive results. However, our study has potential limitations, including small sample size (which partly due to the rarity of MS itself) and a relatively short follow-up period. Since it is a cohort study, dropout was inevitable. Data regarding disease duration and relapse frequency were obtained through self-reporting which possibly contributed to recall bias. We also did not analyze factors affecting disability, such as the treatments received during the study. Lastly, our study needs further statistical analysis with Generalized Estimating Equations (GEEs) to identify the correlation between disease duration, the number of relapses, EDSS, and the structure and function of the optic nerve.

## Figures and Tables

**Table 1:** Demographic and clinical data of multiple sclerosis (MS), healthy controls for OCT (HC OCT), and healthy controls for VEP (HC VEP).

Variable	MS (*N* = 32)	HC OCT (*N* = 22)	*p*	HC VEP (*N* = 110)	*p*
*N (%)*					
*Sex; N (%)*					
(i) Male	5 (15.6%)	5 (22.7%)	0.72^a^	55 (50%)	**0.0005** ^**c**^
(ii) Female	27 (84.4%)	17 (77.3%)		55 (50%)	
*Median (range)*					
Age (years)	29.5 (19.0–59.0)	28.5 (20.0–57.0)	0.42^b^	29.0 (19.0–55.0)	0.24^b^
Disease duration (years)	3.72 (0.50–15.94)	—	—	—	
Relapse (*n*)	4.0 (0.0–13.0)	—	—	—	
EDSS	3.0 (0.0–8.0)	—	—	—	

EDSS: Expanded Disability Status Scale. ^a^Fisher's test. ^b^Mann–Whitney test. ^c^Chi-square test.

**Table 2:** Comparison of BCVA, contrast sensitivity, GCIPL, RNFL, P100 latency, and fundus image between MS and healthy controls (HCs).

Variable	*n*	MS	*n*	HC	*p* value
*Median (range)*					
**BCVA (logmar)**	58	0.00 (0.00–1.77)	44	0.00 (0.00–0.10)	**0.001** ^**b**^
**Contrast sensitivity**	53	1.65 (0.90–1.95)	44	1.95 (1.65–1.95)	**0.00000000028** ^**b**^

Mean ± standard deviation					
*GCIPL*					
Mean	54	70.89 ± 10.60	43	84.72 ± 2.99	**0.00000000000036** ^**a**^
Superior	54	71.11 ± 11.44	43	86.05 ± 2.96	**0.000000000083** ^**b**^
Superonasal	54	71.00 ± 12.51	43	87.58 ± 3.56	**0.000000000033** ^**b**^
Inferonasal	54	70.28 ± 12.74	43	85.53 ± 3.99	**0.0000000027** ^**b**^
Inferior	54	69.35 ± 11.06	43	82.30 ± 4.07	**0.000000000022** ^**a**^
Inferotemporal	54	71.83 ± 10.12	43	83.72 ± 3.74	**0.0000000021** ^**b**^
Superotemporal	54	71.15 ± 9.82	43	83.53 ± 4.00	**0.00000000006** ^**b**^

*RNFL*					
Mean	54	88.48 ± 14.08	44	102.50 ± 7.76	**0.000000016** ^**a**^
Superior	54	115.69 ± 21.95	44	132.36 ± 12.34	**0.000008** ^**a**^
Nasal	54	68.17 ± 10.60	44	71.73 ± 11.58	0.12^a^
Inferior	54	110.28 ± 20.78	44	134.93 ± 15.14	**0.00000000099** ^**a**^
Temporal	54	59.37 ± 16.82	44	70.80 ± 7.90	**0.00003** ^**a**^
**P100 latency**	54	116.63 ± 19.19	220	100.62 ± 5.73	**0.0000000098** ^**b**^

*Fundus image; N (%)*					
Normal	31 (57.4)		44 (100)		**0.00000075** ^**c**^
Optic atrophy	23 (42.6)		0 (0,0)		

BCVA: best-corrected visual acuity; GCIPL: ganglion cell inner plexiform layer; RNFL: retinal nerve fiber layer. ^a^T-independent test, ^b^Mann–Whitney test, ^c^Chi-square test.

**Table 3:** BCVA, contrast sensitivity, and P100 latency value of MS eyes at baseline, 6 months, and 12 months.

Visual parameter	MS						*p*
	*n*	Baseline	*n*	6 months	*n*	12 months	
BCVA (logmar)	58	0.0 (0.00–1.77)	34	0.00 (0.00–1.85)	20	0.00 (0.00–1.78)	0.50^a^
							0.61^b^
							0.23^c^

Contrast sensitivity	53	1.65 (0.90–1.95)	33	1.65 (1.05–1.95)	19	1.65 (1.20–1.95)	0.51^a^
							0.59^b^
							0.16^c^

P100 latency	54	116.63 ± 19.19	33	110.07 ± 19.97	19	124.43 ± 19.13	0.14^a^
							**0.00023** ^b^
							**0.002** ^c^

BCVA= best-corrected visual acuity. ^a^0 vs 6 months, ^b^6 vs 12 months, ^c^0 vs 12 months. The Friedman test was followed by the Wilcoxon test.

**Table 4:** Comparison of baseline, 6 months, and 12 months of evaluation on GCIPL and RNFL thickness of MS eyes.

Variable	MS						*p*		
	*n*	Baseline	*n*	6 months	*n*	12 months	0 vs 6	6 vs 12	0 vs 12
*Mean* *±* *standard deviation*									
*GCIPL*									
Mean	54	70.89 ± 10.60	34	69.38 ± 10.87	20	67.95 ± 11.12	0.105^a^	0.150^a^	**0.007** ^a^
Superior	54	71.11 ± 11.44	34	70.32 ± 11.07	20	68.82 ± 11.59	1.000^b^	0.579^b^	0.619^b^
Superonasal	54	71.00 ± 12.51	34	69.88 ± 12.65	20	68.05 ± 12.90	0.294^a^	**0.038** ^a^	0.053^a^
Inferonasal	54	70.28 ± 12.74	34	68.65 ± 12.65	20	67.41 ± 13.75	**0.039** ^a^	0.495^a^	**0.012** ^a^
Inferior	54	69.35 ± 11.06	34	68.18 ± 11.02	20	66.45 ± 11.68	0.394^a^	0.208^a^	**0.046** ^a^
Inferotemporal	54	71.83 ± 10.12	34	70.06 ± 10.46	20	68.36 ± 10.49	0.108^a^	0.284^a^	**0.012** ^a^
Superotemporal	54	71.15 ± 9.82	34	69.15 ± 10.25	20	69.00 ± 10.37	0.080^b^	1.000^b^	0.389^b^

*RNFL*									
Mean	54	88.48 ± 14.08	34	87.62 ± 14.12	20	86.50 ± 14.22	1.000^b^	1.000^b^	0.836^b^
Superior	54	115.69 ± 21.95	34	115.91 ± 25.69	20	114.50 ± 22.71	1.000^b^	1.000^b^	1.000^b^
Nasal	54	68.17 ± 10.60	34	67.79 ± 9.66	20	68.50 ± 8.77	0.933^a^	0.093^a^	**0.004** ^a^
Inferior	54	110.28 ± 20.78	34	107.62 ± 22.09	20	105.45 ± 22.25	1.000^a^	0.368^a^	0.727^a^
Temporal	54	59.37 ± 16.82	34	59.32 ± 15.61	20	57.85 ± 17.41	0.376^b^	0.537^b^	1.000^b^

*Gambaran fundus; N (%)*									
Normal	31 (57.4)	—	14 (41.2)	—	8 (36.4)	—	0.138^c^	0.719^c^	0.096^c^
Atrofi papil	23 (42.6)	—	20 (58.8)	—	14 (63.6)	—			

GCIPL: ganglion cell inner plexiform layer; RNFL: retinal nerve fiber layer, ^a^Friedman test followed by Wilcoxon test, ^b^Repeated ANOVA followed by Bonferonni test, ^c^Chi-square test.

**Table 5:** Comparison of GCIPL and RNFL thickness between RRMS and SPMS eyes.

Variable	*n*	RRMS	*n*	SPMS	*p*
*Mean* *±* *standard deviation*					
*GCIPL*					
Mean	42	73.19 ± 10.32	12	62.83 ± 7.31	**0.002** ^**a**^
Superior	42	73.64 ± 10.96	12	62.25 ± 8.50	**0.002** ^**b**^
Superonasal	42	73.81 ± 11.98	12	61.17 ± 9.16	**0.002** ^**a**^
Inferonasal	42	73.14 ± 12.56	12	60.25 ± 7.26	**0.003** ^**a**^
Inferior	42	71.98 ± 10.95	12	60.17 ± 4.93	**0.001** ^**a**^
Inferotemporal	42	73.98 ± 9.96	12	64.33 ± 6.72	**0.003** ^**a**^
Superotemporal	42	73.29 ± 9.22	12	63.67 ± 8.32	**0.002** ^**b**^

*RNFL*					
Mean	42	91.79 ± 13.88	12	76.92 ± 7.00	**<0.001** ^**b**^
Superior	42	119.43 ± 22.02	12	102.58 ± 16.50	**0.018** ^**b**^
Nasal	42	69.33 ± 11.05	12	64.08 ± 7.93	0.132^b^
Inferior	42	115.88 ± 19.64	12	90.67 ± 10.22	**<0.001** ^**a**^
Temporal	42	61.93 ± 17.42	12	50.42 ± 10.93	**0.035** ^**b**^

GCIPL: ganglion cell inner plexiform layer; RNFL: retinal nerve fiber layer, RRMS: relapsing-remitting multiple sclerosis; SPMS: secondary progressive multiple sclerosis. ^a^Mann–Whitney test, ^b^T-independent test.

**Table 6:** Profile of MS eyes that developed optic atrophy at 6 months based on the history of ON.

Eyes code	024	069	071	072	113	114
History of ON	Yes	Yes	No	No	No	No

Sex	Female	Female	Female	Female	Female	Female

Age (years)	29	32	47	47	27	27

MS subtype	RRMS	RRMS	RRMS	RRMS	RRMS	RRMS

Disease duration (years)	6.29	0.75	0.5	0.5	3	3

Number of relapses	6	3	0	0	6	6

EDSS	1.5	3.0	1.0	1.0	6.0	6.0

BCVA (logmar)	0.00	0.00	0.00	0.00	0.00	0.00

Contrast sensitivity	1.65	1.95	1.65	1.65	1.95	1.95

P100 latency	115.30	103.15	104.30	110.85	163.60	125.60

*GCIPL*						
Mean	62.00	84.00	78.00	80.00	72.00	75.00
Superior	65.00	87.00	77.00	79.00	73.00	73.00
Superonasal	66.00	87.00	80.00	80.00	73.00	72.00
Inferonasal	64.00	82.00	80.00	84.00	75.00	73.00
Inferior	56.00	82.00	77.00	79.00	69.00	78.00
Inferotemporal	60.00	85.00	76.00	81.00	71.00	78.00
Superotemporal	64.00	82.00	75.00	75.00	69.00	74.00

*RNFL*						
Mean	79.00	106.00	93.00	96.00	93.00	90.00
Superior	115.00	132.00	126.00	132.00	106.00	110.00
Nasal	62.00	71.00	61.00	68.00	68.00	73.00
Inferior	93.00	143.00	123.00	128.00	109.00	108.00
Temporal	46.00	78.00	61.00	55.00	87.00	71.00

ON: optic neuritis; EDSS: Expanded Disability Status Scale.

## Data Availability

The data that support the findings of this study are available from the corresponding author upon reasonable request.
